# Chinese expert consensus on the diagnosis and treatment of bone metastasis in lung cancer (2022 edition)

**DOI:** 10.1016/j.jncc.2023.08.004

**Published:** 2023-08-19

**Authors:** Jianchun Duan, Wenfeng Fang, Hairong Xu, Jinliang Wang, Yuan Chen, Yi Ding, Xiaorong Dong, Yun Fan, Beili Gao, Jie Hu, Yan Huang, Cheng Huang, Dingzhi Huang, Wenhua Liang, Lizhu Lin, Hui Liu, Zhiyong Ma, Meiqi Shi, Yong Song, Chuanhao Tang, Jialei Wang, Lifeng Wang, Yongfeng Wang, Zhehai Wang, Nong Yang, Yu Yao, Yan Yu, Qitao Yu, Hongmei Zhang, Jun Zhao, Mingfang Zhao, Zhengfei Zhu, Xiaohui Niu, Li Zhang, Jie Wang

**Affiliations:** 1 CAMS Key Laboratory of Translational Research on Lung Cancer, State Key Laboratory of Molecular Oncology, Department of Medical Oncology, National Cancer Center/National Clinical Research Center for Cancer/Cancer Hospital, Chinese Academy of Medical Sciences, Peking Union Medical College, Beijing, China; 2 Department of Respiratory Medicine, Shanxi Province Cancer Hospital/Shanxi Hospital Affiliated to Cancer Hospital, Chinese Academy of Medical Sciences/Cancer Hospital Affiliated to Shanxi Medical University, Taiyuan, China; 3 Department of Medical Oncology, State Key Laboratory of Oncology in South China, Collaborative Innovation Center for Cancer Medicine, Sun Yat-sen University Cancer Center, Guangzhou, China; 4 Department of Orthopaedic Oncology Surgery, Beijing Jishuitan Hospital, Capital Medical University, Beijing, China; 5 Department of Oncology and Institute of Translational Medicine, Medical Innovation Research Center and the Fifth Medical Center, Chinese PLA General Hospital, Beijing, China; 6 Department of Oncology, Tongji Hospital of Tongji Medical College, Huazhong University of Science and Technology, Wuhan, China; 7 Cancer Center, Union Hospital of Tongji Medical College, Huazhong University of Science and Technology, Wuhan, China; 8 Department of Thoracic Medical Oncology, The Cancer Hospital of the University of Chinese Academy of Sciences (Zhejiang Cancer Hospital), Hangzhou, China; 9 Department of Thoracic Oncology, Zhejiang Cancer Hospital, Hangzhou, China; 10 Department of Respiratory Medicine, Zhongshan Hospital, Fudan University, Shanghai, China; 11 Department of Medical Oncology, Fujian Cancer Hospital, Fuzhou, China; 12 Department of Thoracic Medical Oncology, Lung Cancer Diagnosis and Treatment Centre, Key Laboratory of Cancer Prevention and Therapy, Tianjin Medical University Cancer Institute and Hospital, National Clinical Research Centre for Cancer, Tianjin, China; 13 Department of Thoracic Surgery/Oncology, the First Affiliated Hospital of Guangzhou Medical University, Guangzhou Institute of Respiratory Disease & Health, Guangzhou, China; 14 Department of Medical Oncology, The First Clinical Medical College, the First Affiliated Hospital of Guangzhou University of Chinese Medicine, Guangzhou, China; 15 Department of Radiation Oncology, Sun Yat-sen University Cancer Center, Guangzhou, China; 16 Department of Medical Oncology, the Affiliated Cancer Hospital of Zhengzhou University/Henan Cancer Hospital, Zhengzhou, China; 17 Department of Medical Oncology, Jiangsu Cancer Hospital, Affiliated Cancer Hospital of Nanjing Medical University, Nanjing, China; 18 Department of Respiratory and Critical Care Medicine, Affiliated Jinling Hospital, Medical School of Nanjing University, Nanjing, China; 19 Department of Oncology, Peking University International Hospital, Beijing, China; 20 Department of Oncology, Fudan University Shanghai Cancer Center, Shanghai, China; 21 Comprehensive Cancer Centre of Drum Tower Hospital, Medical School of Nanjing University, Nanjing, China; 22 Department of Respiratory Medicine, Linyi Central Hospital, Linyi, China; 23 Department of Oncology, Shandong Cancer Hospital, Jinan, China; 24 Department of Medical Oncology, Hunan Cancer Hospital, Affiliated Cancer Hospital of Xiangya School of Medicine, Changsha, China; 25 Department of Oncology Internal Medicine, the First Affiliated Hospital of Xi'an Jiaotong University, Xi'an, China; 26 School of Public Health, Xi'an Jiaotong University, Xi'an, China; 27 Department of Respiratory Oncology, Affiliated Tumor Hospital of Guangxi Medical University, Nanning, China; 28 Department of Clinical Oncology, Xijing Hospital, the Fourth Military Medical University, Xi'an, China; 29 Department of Thoracic Medical Oncology, Peking University Cancer Hospital & Institute, Beijing, China; 30 Department of Medical Oncology, the First Hospital of China Medical University, Shenyang, China; 31 Department of Radiation Oncology, Fudan University Shanghai Cancer Center, Shanghai, China

**Keywords:** Lung cancer, Bone metastasis, Multi-disciplinary therapy, Bone-modifying drugs

## Abstract

Lung cancer is the leading cause of cancer-related deaths worldwide. Bone is a common metastatic site of lung cancer, about 50% of bone metastatic patients will experience skeletal related events (SREs). SREs not only seriously impact the quality of life of patients, but also shorten their survival time. The treatment of bone metastasis requires multi-disciplinary therapy (MDT) and development of individualized treatment plan. In order to standardize the diagnosis and treatment of bone metastasis in lung cancer, the expert group of the MDT Committee of the Chinese Medical Doctor Association has developed the expert consensus on the diagnosis and treatment of lung cancer bone metastasis.

## 1 Introduction

**Fig. 1:**
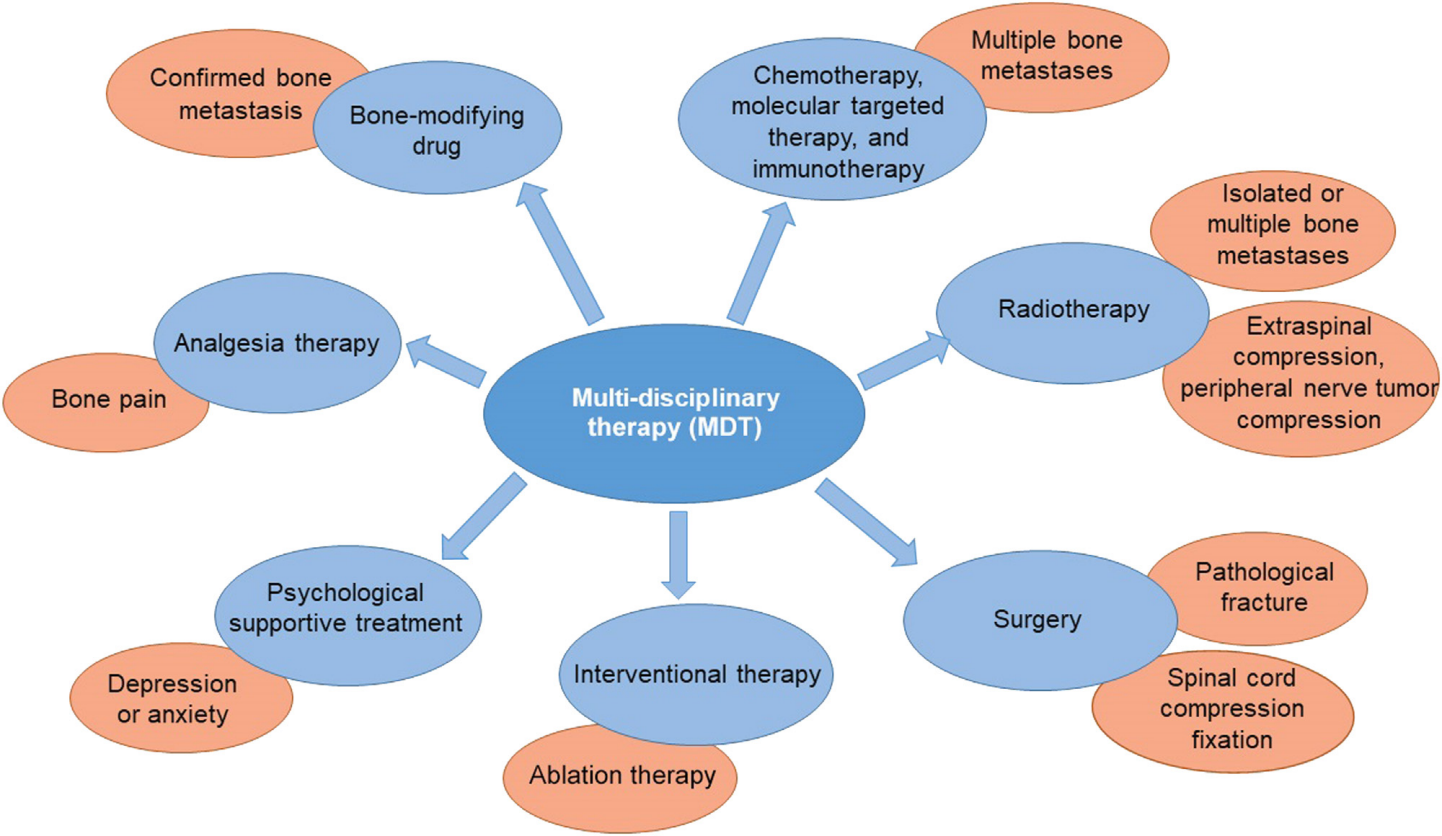
The recommended MDT for bone metastasis in lung cancer. The core of the MDT concept is the collaborative participation of multiple disciplines (e.g., oncology, orthopedics, radiotherapy, etc.) and the establishment of systematic and individualized treatment regimens according to disease conditions.

Lung cancer is the leading cause of cancer-related deaths worldwide, and the majority of patients are diagnosed at advanced stages.^1^, ^2^, ^3^ In China, an estimated 4.06 million new cancer cases occurred in 2016 and lung cancer remained the most common cancer, with an estimated 0.8 million new cases.^4^ Bone is one of the most common sites of hematogenous metastasis of lung cancer, with an incidence of approximately 30–40%.^5^, ^6^, ^7^, ^8^ With the improvement in treatment options, the 5-year survival rate of patients with advanced lung cancer is gradually increasing.^9^^,^^10^ However, there is an increased risk of bone metastasis and skeletal-related events (SREs), such as pathological fracture and spinal cord compression.^7^^,^^11^^,^^12^ Usually, bone metastasis indicated a decline in the quality of life (QOL) and shortened the survival of patients,^13^ while SREs would further affect patients' QOL and increase the overall treatment burden. Therefore, based on the management of primary disease, the active treatment of bone metastasis is particularly important. In order to help patients achieve the greatest benefit in their life expectancy and QOL, it is necessary to establish individualized, comprehensive regimens for bone metastasis in a planned and reasonable way, under the guidance of the multi-disciplinary therapy (MDT) pattern, so as to reduce or delay bone metastasis complications and SREs, as well as ensure the administration of anti-tumor therapies (Fig. 1).

## 2 Consensus development process

The formation process of the expert consensus mainly included the following steps: the formation of the expert group, the presentation of clinical problems, expert discussions, and expert voting decisions. Clinical problems recognized by over 75% of the experts would be recommended in the expert consensus.

The expert group included experts from the departments of oncology, orthopedics, and radiotherapy. The presentation of clinical problems was completed offline by the expert group members. Finally, the components of this consensus were determined to be epidemiology, pathogenesis, diagnosis, and treatment. Due to the impact of the COVID-19 pandemic, all three rounds of expert discussions were conducted online.

## 3 Pathologic characteristics and pathogenesis of bone metastasis in lung cancer

The most frequent site of bone metastasis in lung cancer is the spine (> 50%), followed by the rib ( about 50%), pelvis (20%), femur (about 15%), and sternum (about 15%).^7^^,^^14^, ^15^, ^16^ Patients with lung adenocarcinoma have a relatively higher risk of bone metastasis compared with other pathological types. Meanwhile, the most common site of bone metastasis depends on the pathological types; it is estimated that approximately 79% of lung adenocarcinoma patients will have spinal metastasis.^17^

According to the characteristics of the lesions, bone metastasis can be divided into the following three types: osteolytic, osteogenic, and mixed type. ^18^ Osteolytic bone metastasis accounts for the majority of lung cancer bone metastasis, with a higher risk of SREs compared with the other types.^19^^,^^20^ The receptor activator of NF-κB (RANK) and its ligand (RANKL) signaling play an important role in the mechanism of osteolytic bone metastases. Tumor cells secrete parathyroid hormone-related peptides (PTHrP) which promote the overexpression of RANKL in osteoblasts. Then, RANKL binds the RANK receptor on osteoclast precursors and stimulates bone destruction and osteolysis by inducing the differentiation, maturation, and activation of osteoclasts. In turn, the process of bone resorption releases factors that promote tumor growth, creating a “vicious circle”.^21^^,^^22^ Some tumor cells may also produce factors that can increase the activity of osteoblasts, including bone morphogenetic proteins (BMPs), transforming growth factor β (TGF-β), endothelin-1, and fibroblast growth factors (FGFs), enhancing the proliferation and differentiation of osteoblasts.^19^ Notably, the pathologic ossification with disordered structures is the end result of osteoblastic bone metastasis, which has a poor mechanical property and a risk of pathological fracture.

## 4 Clinical features

Approximately 50% of lung cancer patients with bone metastasis might experience bone complications, including bone pain, pathological fracture, spinal cord compression, and hypercalcemia.^23^ Among these, bone pain is the most frequent symptom of bone metastasis. The over-activated osteoclasts can dissolve bone minerals and release massive hydrogen ions (H^+^) to stimulate peripheral pain receptors, which is one of the direct causes of bone pain. Meanwhile, severe pain is not only caused by the pain factos produced by tumor cells, such as prostaglandin, interleukin-1 (IL-1), and the tumor necrosis factor (TNF), but also caused by the tumor involvement in the periosteum, nerve, and soft tissue. Pathological fracture often affects patients’ self-mobility and QOL seriously. Besides, spinal cord compression may occur in patients with vertebral metastasis due to the tumor's direct compression or the related vertebral fracture. These patients usually manifest impairments of extremity sensory and muscle strength, or even paraplegia in serious cases. Hypercalcemia is graded into three severity categories: mild, moderate, and severe, according to the levels of serum calcium; of these, severe persistent hypercalcemia is one mortality cause in lung cancer patients with bone metastasis. Besides, lung cancer patients with bone metastasis at an advanced stage may also report fatigue, emaciation, anemia, low-grade fever, and other symptoms. Although there are some associations between clinical features of bone metastasis and SREs, they are not completely equivalent. SREs were defined as events that indicate the progression of bone metastases. Nevertheless, since pathological fracture and spinal cord compression can be evaluated objectively, they could be regarded as either clinical features or SREs. It is estimated that 22%−59% of lung cancer patients with bone metastasis have a risk of SREs.^24^

Due to the physical and psychological pressure caused by both the primary tumor and the bone metastasis, lung cancer patients with bone metastasis might show some symptoms, such as anxiety, depression, disappointment, and loneliness. If these symptoms are not well-controlled, they may affect the confidence of patients in continuing antitumor therapy.

## 5 Diagnosis

Lung cancer is often a latent disease; thus, most patients are diagnosed at an advanced stage. Generally, bone metastases occurred at diagnosis in more than half of the patients and appeared in others at approximately nine months of follow-up.^25^ Thus, the risk of bone metastasis in lung cancer patients should be closely monitored. Once patients with confirmed lung cancer develop clinical manifestations (such as bone pain, pathological fracture, elevated alkaline phosphatase [ALP], continuously elevated levels of tumor biomarkers without lesion progression, spinal cord or nerve compression, or hypercalcemia), further imaging examinations, such as X-ray, computed tomography (CT), magnetic resonance imaging (MRI), etc., should be performed on the painful site, symptomatic site, and spinal cord compression site. Notably, for patients who have the first onset as orthopedic symptoms, it is necessary to detect the primary lesion actively and evaluate the complexity in obtaining the pathological tissue of bone metastases, so that we could identify the pathologic type by the selective biopsy of the bone, lung, or other metastases.

**Fig. 2:**
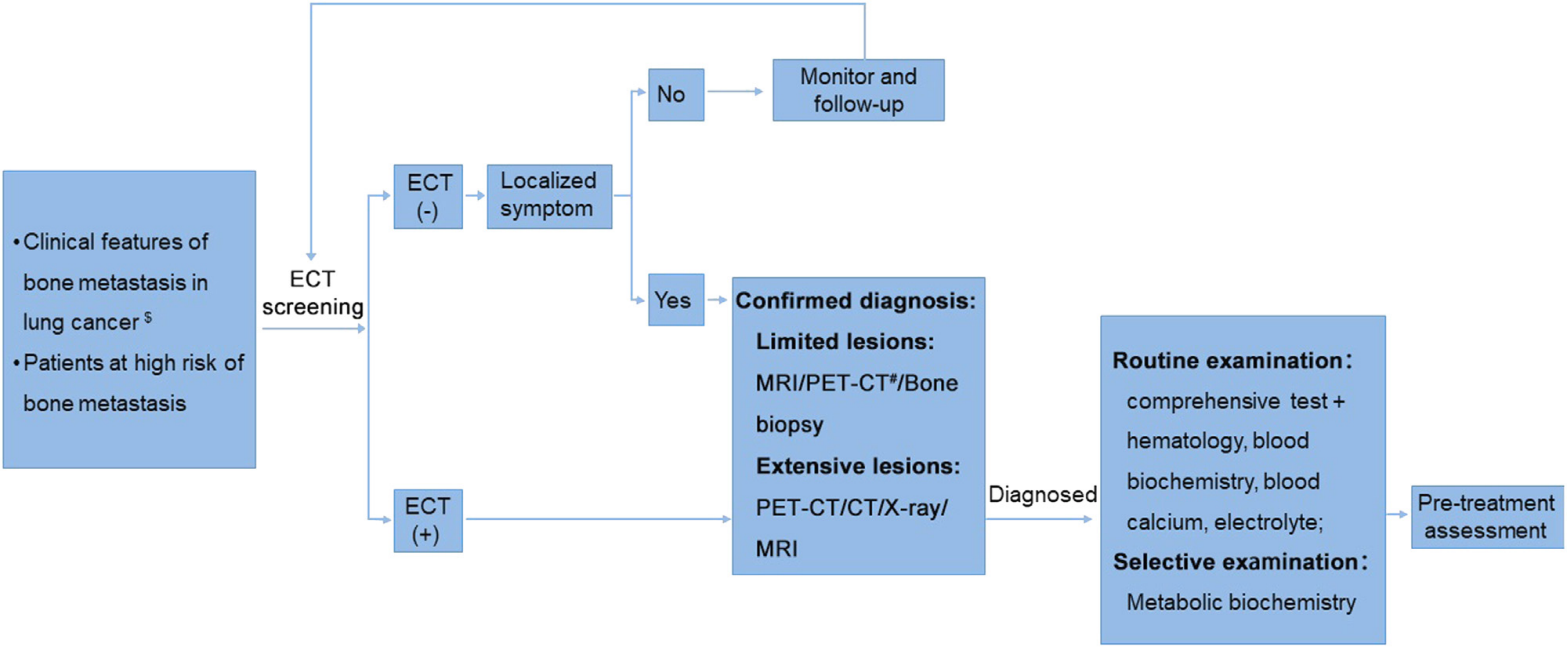
The recommended diagnosis flow of bone metastasis in lung cancer. ^$^ Clinical features of bone metastasis: bone pain, limitation of motion, pathological fractures, spinal cord compression, spinal nerve compression, hypercalcemia, etc. ^#^ PET-CT is not recommended as a routine test due to its high cost. CT, computed tomography; ECT, emission computed tomography; MRI, magnetic resonance imaging; PET-CT, positron emission computed tomography.

Usually, imaging examination is sensitive to osteolytic lesions, but has poor sensitivity to osteogenic lesions. Even so, imaging examination is useful in determining the range of osteogenic lesions. The recommended diagnosis flow of bone metastasis in lung cancer is shown in Fig. 2.

### 5.1 Imaging diagnosis

#### 5.1.1 X-ray

X-ray is currently the most basic and valuable diagnostic tool for cancer bone metastasis. Osteolytic lesions are the most common type of bone metastasis in lung cancer, observed from X-ray images. It is generally acknowledged that bone destruction may not be detectable by X-ray until more than 30%−50% of the vertebral body has been destroyed. Osteoblastic changes can be used as one indicator to identify the efficacy of medical treatment.^26^ Nevertheless, the plain radiographs has poor sensitivity in detecting early bone metastasis.^10^^,^^26^ When intramedullary metastasis did not involve the bone cortex, lesions would be covered up by the high-density cortex, leading to missed diagnosis. Therefore, bone metastasis usually appear on the X-ray films 3–6 months later than that on the emission computed tomography (ECT). Currently, X-ray is often used as the supplementary assessment for symptomatic sites or abnormalities detected by other imaging examinations,^27^ or used as the routine method of follow-up examination.

#### 5.1.2 CT/enhanced CT

CT scan has more sensitivity than X-ray in detecting bone metastasis. CT scan thus has emerged as a practical tool for the diagnosis of bone metastasis and the evaluation of bone destruction. CT scan shows accurately the bone destruction and surrounding soft tissue masses. Meanwhile, the enhanced CT is useful in detecting the blood supply of bone metastasis, as well as the relationship between lesions and peripheral nerves or vascular structures. Besides, CT scan can detect bone metastasis in patients who present positive results in whole-body bone scanning but negative on the X-ray film, and patients with localized symptoms, suspected bone metastasis, or contraindications to MRI.

#### 5.1.3 MRI

MRI has high sensitivity but low specificity for the diagnosis of bone metastasis. MRI can accurately reveal the location and range of the metastatic lesions, as well as the condition of surrounding soft tissue and spinal cord compression by multi-plane and multi-sequence imaging. Moreover, the contrast-enhanced MRI is well suited to detect more bone metastasis sites. MRI is superior to whole-body bone scanning in diagnostic sensitivity, particularly for early-stage intramedullary metastases. Thus, MRI is the preferred diagnostic method for intramedullary spinal cord metastasis. The whole-body MRI scanning recently overcame the limitation of conventional MRI in the scan range and presented a similar sensitivity to positron emission tomography/CT (PET/CT) in the diagnosis of bone metastasis.^28^^,^^29^ In addition, PET/MRI shows potential value in the diagnosis of bone metastasis.^30^^,^^31^

#### 5.1.4 Single-photon emission computed tomography

Single-photon emission computed tomography (SPECT, also known as ECT) is preferred to screen bone metastasis, as it can identify the osteolytic, osteogenic, and mixed bone metastases in the early stage of the disease. Especially, SPECT has superiority in the detection of osteogenic metastasis, due to the advantages of high sensitivity, whole-body bone imaging, and rare misdiagnosis. However, in addition to bone metastases, other bone lesions can also demonstrate nuclide enrichment and then lead to false-positive results. Therefore, SPECT has poor specificity in the diagnosis of bone metastasis. The whole-body bone scanning is recommended for the tumor staging in the confirmation of lung cancer. A baseline bone scan combined with regular bone monitoring (once or twice annually) would achieve the dynamic comparison, which is clinically meaningful in the diagnosis of bone metastasis.

#### 5.1.5 PET/CT

PET/CT has high sensitivity (62%−100%) and specificity (96%−100%) for the detection of bone metastasis.^32^, ^33^, ^34^ Of these, ^18^F-fluorodeoxyglucose (^18^F-FDG) PET/CT is the most sensitive to osteolytic and bone marrow metastasis, and ^18^F-sodium fluoride (^18^F-NaF) PET/CT is the most sensitive to osteogenic metastasis. The appropriate photographic developer is particularly important for the diagnosis of unifocal metastases. ^18^F-FDG PET/CT is reported to not only show the skeletal involvement of the whole body, but also evaluate the tumor staging. Nevertheless, it is associated with high cost.^35^ The novel imaging equipment PET/MR is an integrated technology with multiple advantages of PET and multiparametric MRI. It has demonstrated superiority to PET/CT with regards to the detection of more, smaller, or earlier bone metastases. Unfortunately, the potency of its clinical application remains to be investigated due to the high cost and poor universality.

Among these imaging methods,^8^, ^9^, ^10^, ^11^, ^12^, ^13^, ^14^ ECT is a primary screening examination, and ECT-positive sites are further confirmed with X-ray, CT and/or MRT scan. Among them, the plain film X-ray is used to reveal the overall bone intensity, while CT is used as diagnosis assistance to assess the range of bone destruction. Besides, MRI evaluates the extent of tumor lesions and spinal cord compression.

### 5.2 Bone biopsy

Most patients with confirmed lung cancer and typical imaging manifestations of bone metastasis can be directly diagnosed. However, a bone biopsy is required for the following indications: (1) Patients with orthopedic symptoms as the first manifestation of lung cancer and difficulty in sampling intrapulmonary lesions; (2) Bone biopsy is required for patients with confirmed lung cancer and isolated bone destruction lesion, since 15%−18% of new bone lesions may be caused by other tumors or non-tumor lesions, but not by bone metastasis in lung cancer; (3) The diagnosis of bone lesions has decisive influence on the therapeutic strategies; (4) Due to the tumor heterogeneity, a bone biopsy remains needed for pathological or molecular typing to guide individualized treatment so as to achieve the optimization of therapeutic strategies.

Precautions for bone biopsy are as follows^16^: (1) Enhanced CT or MR scan should be performed before puncture biopsy to avoid the necrotic area and sampling osteolytic area possible, so as to meet the criteria of pathological and molecular diagnosis; (2) A bone biopsy for definitive diagnosis of bone metastasis should be performed before any treatment; (3) The bone biopsy typically does not lead to pathological fracture; (4) Biopsy of bone metastases should follow the principles for the biopsy sampling of musculoskeletal tumors.

### 5.3 Biomarkers of bone metabolism

Biomarkers of bone metabolism reflect the rate of bone resorption and formation and also indicate the degree of bone destruction and repair during the process of bone metastasis. Currently, they have emerged as a potential new technique for the diagnosis and monitoring of disease progression. However, the clinical application of biomarkers (except for ALP) remains in the exploratory stage.^36^^,^^37^ At present, the recognized clinical biomarkers include N-telopeptides of type I collagen (NTX), C-telopeptide of type I collagen (CTX), and bone alkaline phosphatase (BALP).^38^, ^39^, ^40^

## 6 Treatment

The goal of treatment for bone metastasis is to prevent or delay SREs, relieve pain and psychological distress, improve the QOL, and prolong life expectancy. As bone metastasis in lung cancer is a systemic disease, comprehensive treatment under the guidance of MDT pattern is recommended, including systematic treatment (chemotherapy, targeted therapy, or immunotherapy) for primary lung cancer, bone-modifying drugs, radiotherapy, surgery, analgesic therapy, psychological supportive treatment, etc.

The therapeutic principle is that systemic treatment is the mainstay of bone metastasis. Among that, immunotherapy, target therapy, and chemotherapy are used for lung cancer. Meanwhile, bone-modifying drugs are used to reduce the risk of SREs, treat hypercalcemia, relieve pain, and improve patients' QOL. In addition, appropriate localized therapies, such as surgery or radiotherapy, can provide more benefit for the management of metastasis-related symptoms, among which surgery is recommended for isolated bone lesions. Besides, symptomatic analgesic therapies can obviously improve patients' QOL. Overall, it is necessary to establish individualized, comprehensive regimens for bone metastasis in a planned and reasonable way, under the guidance of the MDT pattern, by considering factors such as individual situations, tumor pathological types and molecular subtypes, the extent of lesion involvement (clinical stages), and disease progression rate.

### 6.1 Efficacy evaluation of bone metastasis in lung cancer

The efficacy evaluation of bone metastasis in lung cancer should be based on comprehensive information about the clinical manifestations, imaging, and tumor markers. Systemic therapy is effective for both primary lung cancer and bone metastasis. The improvement of clinical symptoms and the decrease of tumor markers usually predict the effectiveness of anti-tumor therapies. However, one situation needs to be carefully distinguished, that is, the type of intraspinal bone metastases can transform from osteolytic lesions to osteogenic lesions after effective treatment, which might in turn aggravate symptoms due to local compression. Comprehensive assessment have to be made based on the whole-body bone scan/PET-CT, X-ray, CT, and even MRI. In the X-ray or CT findings, the transition from osteolytic lesions to osteogenic lesions often indicates the effectiveness of anti-tumor therapies. As the dynamic change is more meaningful for the efficacy evaluation, the collection of pre-treated images and data should be taken seriously. For hospitals that have no MDT team, it is recommended to consult a specialist team, so as to obtain expert opinions on the therapeutic regimens or efficacy evaluation.

### 6.2 Anti-tumor treatment

Systemic therapy, including immunotherapy, targeted therapy, and chemotherapy, is the cornerstone of the treatment for advanced lung cancer. The specific therapeutic regimen should be established according to the regularly updated guidelines, including the National Comprehensive Cancer Network (NCCN) Guidelines for Non-Small Cell Lung Cancer (NSCLC),^41^ the European Society for Medical Oncology (ESMO) Clinical Practice Guidelines for Metastatic NSCLC,^42^ and the Chinese Society of Clinical Oncology (CSCO) Clinical Guidelines for the Diagnosis and Treatment of NSCLC.^43^

#### 6.2.1 Immunotherapy

Immunotherapy is the main treatment option for patients with driver-gene-negative advanced lung cancer. The anti-PD-1 antibodies could bind the PD-1 receptor in T cells, while the anti-PD-L1 antibodies bind the PD-L1 receptor in immune cells or tumor cells, so as to block the inhibitory effect of the PD-1/PD-L1 pathway on T cells and eventually activate the anti-tumor activity. According to guidelines on lung cancer treatment, the combination of anti-PD-1/PD-L1 blockade with chemotherapy is preferred as the first-line treatment for driver-gene-negative advanced NSCLC with PD-1/PD-L1 low or negative expression; meanwhile, anti-PD-1/PD-L1 monotherapy is recommended for NSCLC with PD-1/PD-L1 high expression. Besides, immune checkpoint inhibitors (ICIs), such as nivolumab and pembrolizumab, can be recommended as the second-line treatment option for patients with disease progression after chemotherapy. The results of clinical trials have demonstrated that anti-PD-1/PD-L1 blockade as the second-line treatment could prolong the median overall survival (OS) of patients with advanced NSCLC.^44^ Reassuringly, several trials on the combination of immunotherapy with RANKL inhibitors in NSCLC are ongoing.^45^

#### 6.2.2 Target therapy

Targeted therapy for lung cancer is one type of the biotherapy model, which targets the driver genes involved in cell canceration or tumor angiogenesis signaling pathways. It can block tumor signaling pathways at the molecular level, thereby inhibiting tumor cell growth, inducing apoptosis, and even inducing complete regression. Notably, compared to patients with non-adenocarcinoma, those with lung adenocarcinoma harbor higher incidences of driver gene mutations, which may be largely benefited from the target therapy.

The target drugs for NSCLC can be divided into several categories according to the drug targets: (1) Epidermal growth factor receptor (EGFR)-tyrosine kinase inhibitors (TKIs), including gefitinib, erlotinib, osimertinib, afatinib, dacomitinib, almonertinib or furmonertinib; (2) TKIs which target echinoderm microtubule-associated protein-like 4-anaplastic lymphoma kinase (EML4-ALK) fusion gene or reactive oxygen species proto-oncogene 1 (ROS1) kinase domain, including crizotinib, alectinib, ceritinib, or ensartinib; and (3) Vascular endothelial growth factor receptor (VEGFR) inhibitors, such as bevacizumab (a humanized anti-VEGF monoclonal antibody) and anlotinib (a third-line treatment drug).

#### 6.2.3 Chemotherapy

Platinum-containing doublet chemotherapy is the standard treatment for advanced driver-gene-negative NSCLC, which was demonstrated to have superiority to monotherapy in terms of objective response rate (ORR) and OS. Among them, cisplatin or carboplatin-based doublet chemotherapy is preferred for lung cancer patients with bone metastasis.

### 6.3 Bone-modifying drugs

Denosumab and bisphosphonates are recommended for the treatment of patients with bone metastasis in lung cancer. Bone-modifying drugs can prevent or delay the occurrence of SREs, and provide benefits for patients with confirmed bone metastasis, regardless of clinical symptoms. Protocol and precautions of bone-modifying drugs are listed in Table 1.

**Table 1:** Protocol and precautions of bone-modifying drugs (initiation time, treatment duration, precautions).

Drug name	Denosumab	Zoledronic acid	Ibandronate sodium	Incadronate disodium
Recommended initial dose and protocol	120 mg, SC on the upper arm, upper thigh, or abdomen, repeat every 4 weeks	4 mg, IV > 15 min, repeat every 3–4 weeks	6 mg, IV > 15 min, repeat every 3–4 weeks; loading therapy: 6 mg/day, IV > 15 min for 3 consecutive days, thereafter repeat every 3–4 weeks (6 mg, IV > 15 min once)	For most patients, ≤10 mg once; for patients > 65 years, 5 mg once (recommended); IV drip for 2–4 h every 3–4 weeks after dissolving in 500–1000 ml normal saline
Instructions	Bone-modifying drugs are recommended for patients with radiographic bone destruction or metastasis if there are no contraindications, but they are not recommended for patients with a risk of bone metastasis alone but undiagnosed. Therefore, relevant examinations before the drug application should include the above imaging examinations and serum markers, such as SPECT, PET-CT, MRI, ALP, and NTX.			
Treatment duration	Bone-modifying drugs are recommended for patients with a life expectancy of at least 3 months, and the treatment duration is recommended at 18 to 24 months depending on the patients' tolerance and benefit. The total treatment duration may be prolonged by extending the treatment interval according to clinical judgment.			
Precautions	1. When using bone-modifying drugs, the risk of SREs should be closely monitored, especially for patients with risk factors; if SREs occur during the treatment, bone-modifying drugs are still recommended to continue if they could reduce the risk of SREs recurrence. 2. Calcium (500 mg/day) and vitamin D (400 IU/day) are recommended when using bone-modifying drugs. 3. Considering the possible ADRs related to bone-modifying drugs, the pre-existing hypocalcemia should be corrected before the administration of bone-modifying drugs. It is recommended to perform an appropriate oral examination cooperating with the stomatology department in advance and pay attention to various factors (e.g. serum calcium, serum creatinine, phosphate, magnesium, renal function, etc.) to avoid invasive operations; during administration, it is suggested to closely monitor patients' health and adjust the treatment regimens according to the patients' conditions to maximize the medication safety.			

Abbreviations: ADR, adverse reactions; ALP, alkaline phosphatase; MRI, magnetic resonance imaging; NTX, N-terminal telopeptide of type 1 collagen; SC, subcutaneous injection; SPECT, single-photon emission computed tomography; SRE, skeletal-related events; PET-CT, positron emission computed tomography; IV, intravenous injection; IV drip, intravenous drip.

#### 6.3.1 Denosumab

Denosumab is a fully human IgG2 monoclonal antibody targeting human RANKL. It can inhibit the differentiation, maturation, and activation of osteoclast by binding with RANKL. The 244 study is a phase III trial comparing denosumab with zoledronic acid (ZA) for delaying or preventing SREs in patients with bone metastasis in solid tumors (except for breast and prostate) or multiple myeloma, based on conventional anti-tumor therapy. In the 244 study, the subgroup analysis of solid tumors (*n* = 1 597; 811 patients with lung cancer) showed that denosumab significantly delayed the time to first on-study SRE by 6 months compared with ZA (21.4 vs 15.4 months), and also reduced the risk of multiple SREs by 15% compared with ZA (hazard ratio [HR], 0.85 [95% confidence intervals, 0.72–1.00]; *P* = 0.048) .^46^^,^^47^ Besides, the subgroup analysis of lung cancer (small cell lung cancer and NSCLC) revealed that denosumab prolonged the OS by 1.2 months compared with ZA (8.9 vs 7.7 months, HR, 0.80 [95% confidence intervals, 0.67–0.95]; *P* = 0.01). Accordingly, denosumab is recommended by the NCCN and ESMO guidelines for lung cancer patients with bone metastasis. In 2020, denosumab was also approved for the treatment of bone metastasis in solid tumor patients with bone metastasis in China.

#### 6.3.2 Bisphosphonates

Bisphosphonates, as stable analogs of pyrophosphate, can be used as conventional drugs alone or in combination with conventional anti-tumor therapy in the treatment of bone metastases. Bisphosphonates were selectively absorbed by osteoclasts to inhibit the maturation and function of osteoclasts or induce the apoptosis of osteoclasts, and in turn, suppress the bone destruction.

With improvements in the drug structure, pharmacokinetics, and safety of bisphosphonates, the ZA, ibandronate, and incadronate have emerged as the commonly used third-generation bisphosphonate drugs. A previous meta-analysis enrolled 12 trials with 1 767 patients with advanced cancer and revealed that bisphosphonates could reduce SRE incidences, relieve bone metastasis-related pain, delay the progression of bone lesions, and further contribute to an improved survival rate.^48^^,^^49^ Furthermore, a multicenter trial included 198 NSCLC patients with metastatic bone diseases and confirmed that bisphosphonates could prevent and delay SREs regardless of the prior SREs, possibly due to its direct anti-tumor effect. Besides, the study reported no osteonecrosis of the jaw (ONJ) in any patient during ZA administration.^48^^,^^49^

#### 6.3.3 Adverse reactions and safety

Bone-modifying drugs are well-tolerated, and accordingly, few patients discontinued treatment due to adverse reactions (ADRs) in the clinic.^50^ The most common ADRs of denosumab and bisphosphonates are nonspecific influenza-like symptoms, including bone pain, fever, fatigue, chills, and joint or muscle pain. Additionally, there are some rare ADRs, such as ONJ, hypocalcemia, mild injection-site reactions, and asymptomatic, treatment-free decreased plasma phosphate. Actually, the incidence of ONJ caused by different bone-modifying drugs is generally comparable. As the occurrence of ONJ is associated with oral infection (or other risk factors), a comprehensive oral examination is recommended at least every 6 months to achieve early treatment once it occurs. In addition, renal function should be monitored when using bisphosphonates. The dose adjustment of bisphosphonates should be performed if renal dysfunction occurrs. However, no dose adjustment is required for denosumab, because it is not metabolized by the kidney.

### 6.4 Analgesic therapy

Comprehensive analgesic therapy is recommended for the pain management of bone metastases in lung cancer; that is, appropriate analgesic methods should be applied to eliminate pain, prevent and manage adverse reactions, and improve the QOL early, continuously, and effectively, according to patients’ diseases and physical conditions, as well as the sites and characteristics of pain. Currently, analgesic therapy includes drug therapy and non-drug therapy (radiotherapy, surgery, and interventional therapy).^51^

#### 6.4.1 Pain assessment

For lung cancer patients suffering from pain caused by bone metastases, adequate pain assessment is the premise for reasonable and effective analgesic therapy. Pain assessment should follow the routine, quantitative, comprehensive, and dynamic evaluation principles according to the Standards for Cancer Pain Management (version 2018).^52^

#### 6.4.2 Principles of analgesics therapy

The analgesic drug therapy for lung cancer patients with bone metastases should follow the guidelines of the World Health Organization (WHO) three-step analgesic ladder and Standards for Cancer Pain Management (version 2018). Briefly, it is suggested to choose appropriate analgesics or adjuvant agents, doses, and frequency based on individual situation (such as the nature and degree of the pain, ongoing treatment, and concomitant diseases), so as to get the best analgesic effects and fewer ADRs.

#### 6.4.3 Types and precautions of common analgesics

(1) Nonsteroidal anti-inflammatory drugs and acetaminophen

Nonsteroidal anti-inflammatory drugs (NSAIDs) have analgesic and anti-inflammatory effects, mainly including aspirin, ibuprofen, and selective COX-2 inhibitors (celecoxib and etoricoxib). Acetaminophen possesses analgesic and antipyretic properties but is essentially devoid of anti-inflammatory activity. At present, it is often used for the relief of mild pain, or for the moderate to severe pain in combination with opioids.

(2) Opioids

Opioids are the first choice for the treatment of moderate and severe cancer pain. Opioid agonists are recommended for chronic cancer pain. Regarding the administration route, oral is the preferred route of administration for long-term opioid therapy. Meanwhile, transdermal absorption or temporary subcutaneous injection is applicable for patients with definite indications. In addition, patient-controlled analgesia can be allowed when necessary. During the opioid therapy, the dose-titration method, the choice of a maintenance drug, and the ADRs management should be noted and follow the Standards for Cancer Pain Management (version 2018).

(3) Bone-modifying drugs

Bone-modifying drugs, such as denosumab and bisphosphonates, can prevent the occurrence of bone pain by inhibiting the activation of osteoclasts and reducing the H^+^ produced by osteolysis. Moreover, studies have shown that bone-modifying drugs can effectively relieve cancer pain and prolong the time for the pain to worsen.^53^

(4) Adjuvant agents

Adjuvant agents are commonly used for the adjuvant analgesia of neuropathic pain, mainly including anticonvulsants, tricyclic antidepressants, corticosteroids, and N-methyl-d-aspartate receptors (NMDAR) antagonists, and local anesthetics.

#### 6.4.4 Non-drug analgesic therapy

Non-drug analgesia can be adopted under the guidance of the MDT pattern for patients with indications of radiation, surgery, or interventional therapy and severe bone pain that has already affected the QOL (as detailed in the corresponding section). In recent years, transcutaneous electrical nerve stimulation (TENS) or hyperthermia combined with drug or non-drug analgesic therapy has also shown encouraging clinical benefits.^54^^,^^55^

### 6.5 Radiotherapy

Radiotherapy is one effective localized treatment to relieve metastatic pain in lung cancer. It could relieve or eliminate pain, prevent pathological fracture and spinal cord compression, and relieve compression complications, thereby improving the QOL and prolonging survival. Radiotherapy includes external beam radiotherapy (EBRT) and radionuclide therapy.

#### 6.5.1 EBRT

EBRT is the preferred palliative radiotherapy for bone metastases in lung cancer. Regional radiotherapy can quickly and effectively relieve pain caused by bone destruction and soft tissue lesions, and patients usually show pain relief within two weeks from the start of treatment.^56^ EBRT indications include (1) Bone metastases with bone pain that required pain relief and functional recovery. The radiotherapy for this indication is the symptomatic treatment for SREs; (2) Selective prophylactic radiotherapy for weight-bearing bone metastases (e.g., spinal or femoral metastases).^15^^,^^57^ The primary aim of prophylactic radiotherapy is tumor control, and thus it is usually used at higher doses.

The evidence on direct comparison of single fractionation radiotherapy (8 Gy, 12 Gy, or 16 Gy) and conventionally fractionated radiotherapy (30 Gy/10 f) is still lacking in China. According to international evidence and domestic reality, the recommendation for treatment dose and indications are listed in Table 2.

**Table 2:** Treatment regimens and goals of radiotherapy for bone pain according to patients' conditions.

Patient's condition	Treatment regimens	Treatment goals
PS > 2, short life expectancy	Single fractionation radiotherapy	Pain relief
Non-weight-bearing bone metastases	Single fractionation radiotherapy	Pain relief, local control of bone metastases
Weight-bearing bone (spine) metastases	Conventionally fractionated radiotherapy	Local control of bone metastases, pain relief

Abbreviation: PS, performance status.

Patients with soft tissue masses are usually required a higher radiation dose. There are also ongoing studies evaluating the benefit of radiation doses greater than 30 Gy in patients with bone metastases. The subsequent reports of authoritative evidence are worth expecting. With the development of radiotherapy technology, stereotactic body radiotherapy (SBRT) has attracted widespread attention because it can significantly improve the local control rate of patients^58^ and relieve bone pain.^59^ Nevertheless, it has not been popularized due to technical difficulty and high equipment requirements.

#### 6.5.2 Radionuclide therapy

Radionuclide therapy, also known as internal radiation therapy, is a minimally invasive treatment that concentrates bone-seeking radiopharmaceuticals in metastatic sites or bone tumors after intravenous injection, and then relieves the biological pain caused by tumor tissues.^60^, ^61^, ^62^, ^63^, ^64^ Nevertheless, some patients may experience myelosuppression and slow recovery after radionuclide therapy, affecting subsequent systemic treatment (e.g., chemotherapy). Furthermore, internal radiation therapy is mostly used for the treatment of bone metastases in breast or prostate cancer, lacking direct evidence of bone metastases from lung cancer. Therefore, radionuclide therapy is recommended after a strict understanding of indications and the weighing of clinical risks and benefits by imaging and MDT evaluation. At present, ^89^Sr is mostly used for the radionuclide therapy of bone metastases.

### 6.6 Surgery

Bone metastasis from lung cancer often leads to a decrease in bone intensity, which in turn affects patients’ motor system functions.^65^^,^^66^ Hence, the goal of surgical treatment is not only to improve the prognosis after primary lung cancer resection in patients with bone metastases,^67^ but also to restore motor system functions. The basic principle of surgery is immediate and stable bone structure fixation without the need to expect complete bone healing.

#### 6.6.1 Main goals of surgery

The main goals of surgery include: (1) To relieve pain, preserve mobility and function, and improve the QOL; (2) To prevent or delay the occurrence of SREs; (3) To treat SREs. Additionally, whether to consider controlling malignancy progress and prolonging survival as the long-term treatment goal depends on various circumstances.

Surgical methods should be comprehensively determined according to lesion sites, the extent of involvement, and the presence of pathological fractures or not.^68^ Ultimately, surgery is expected to significantly relieve pain, preserve bone and joint functions, and improve the QOL.^69^, ^70^, ^71^

#### 6.6.2 Expert evaluation criteria

**Fig. 3:**
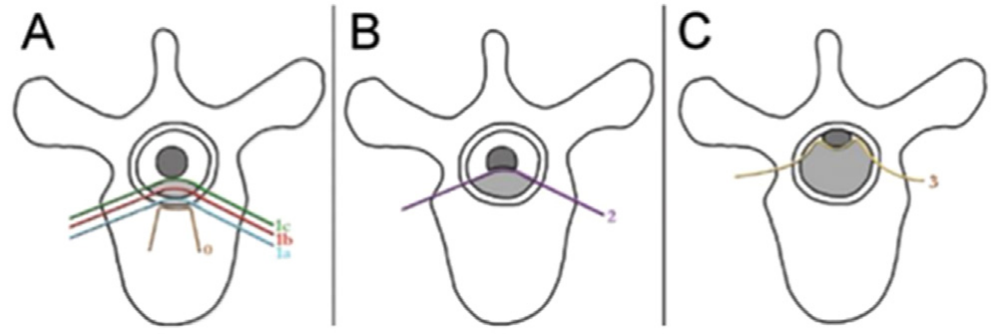
Schematic diagram of ESCC scale. ESCC scale: Grade 0, bone disease alone; Grade 1a, epidural impingement without deformation of the thecal sac; Grade 1b, deformation of the thecal sac without spinal cord abutment; Grade 1c, deformation of the thecal sac with spinal cord abutment, but without cord compression (A). Grade 2, spinal cord compression, but with CSF visible around the cord (B). Grade 3, spinal cord compression, no CSF visible around the cord (C). CSF, cerebrospinal fluid; ESCC, Epidural Spinal Cord Compression.

Bone tumor specialists focus on assessing the probability and consequences of SREs occurrence (e.g., bone pain, pathological fracture, spinal compression fracture, and the risk of spinal nerve compression). The commonly used scoring systems are the Mirels score to assess fracture risk (Table 3) and the Epidural Spinal Cord Compression (ESCC) scale to evaluate spinal cord compression (Fig. 3).^18^

**Table 3:** Mirels score to assess fracture risk*.

Score	Lesion	Size	Site	Pain
1	Osteogenic	< 1/3	Upper limb	Mild
2	Mixed	1/3 - 2/3	Lower limb	Moderate
3	Osteolytic	> 2/3	Pertrochanteric	Severe

⁎ The total Mirels score is 12. Score ≤ 7 indicates a low risk of pathological fracture (4%) and surgery should be not considered; Score = 8, indicates a fracture risk of 15%; Score = 9, indicates a fracture risk of 33%; Score ≥ 9 implies an indication for preventive fixation.

#### 6.6.3 Surgical indications and contraindications

Indications for surgical treatment include: (1) The life expectancy is over 3 months; (2) Pre-conditions are good enough to undergo surgery and anesthesia; (3) Expected better QOL after operation. Construction after surgery must provide enough stability to allow immediate ambulation to facilitate further treatment and care; (4) Patients are expected to have a relatively long tumor-free or progression-free period; (5) Effective systemic treatment but with local symptoms; (6) Isolated bone metastases; (7) Patients with a high risk of pathological fractures; (8) Patients with or at high risk of spinal instability, spinal cord compression.^72^

Contraindications for surgical treatment include: (1) The life expectancy is less than 3 months; (2) Multiple bone destruction; (3) Metastases to multiple organs; (4) Poor physical conditions, with contradictions to surgery.

#### 6.6.4 Timing of surgery

At the following time points sugery may be considered for patients: (1) Patients with a malignancy history appear to have an isolated bone metastasis on radiological and histological examinations; (2) X-ray of weight-bearing bones shows bone destruction; (3) Bone destruction continues to progress after conservative treatment; (4) Bone pain deteriorates after conservative treatment; (5) Motor system function cannot resume after conservative treatment; (6) Patients with pathological fractures; (7) Patients with neural compression symptoms; (8) Patients with spinal osteolysis and a high risk of paraplegia; (9) Metastatic lesions which are not sensitive to radiotherapy and chemotherapy.

### 6.7 Interventional therapy

Minimally invasive interventional therapy has been widely used in the localized management of bone metastasis, pain relief, and the QOL improvement, due to the multiple advantages such as simplicity in operation, minimal invasiveness, safety, and few ADRs, and faster recovery.

Ablation therapy is the most used at present. Ablation therapy is a precise and minimally invasive approach that induces the irreversible damage or coagulative necrosis of tumor cells in lesions by using the thermo-biological effect.^73^ It could effectively relieve pain and improve the QOL.^74^, ^75^, ^76^ High intensity focused ultrasound (HIFU) can concentrate scattered ultrasonic energy to generate instantaneous high temperature and kill tumor cells with thermal, cavitation, and mechanical effects.^77^ Besides, previous studies have shown that HIFU or cryoablation could achieve favorable effects in the treatment of bone metastasis pains.^78^

Osteoplasty is an interventional therapy by injecting polymethyl methacrylate (PMMA, also known as bone cement) into lesions via puncture channels to stabilize the bone structure, relieve pain, and locally control tumors. The osteoplasty mainly includes percutaneous vertebroplasty, kyphoplasty, and the bone perfusion for systemic irregular bone and limb long bone. Osteoplasty can relieve the pain of bone metastatic sites, with a low recurrence rate.^79^, ^80^, ^81^ Among them, percutaneous osteoplasty is suitable for osteolytic primary tumors or bone metastases. The contraindications of osteoplasty include: (1) Severe nervous system diseases or poor physical conditions with difficulty in tolerating surgery and anesthesia; (2) Uncontrolled coagulation dysfunction; (3) Tumor invasion of key organs, nerves, or blood vessels; (4) Active infection; (5) More than five metastases or extensive diffuse metastases.^82^

### 6.8 Supportive treatment

The basic principle of bone metastases therapy is palliative care. Thus, it is suggested to provide supportive treatment and symptomatic treatment for primary tumors, bone metastases, and SREs. Besides, a multidisciplinary team should be established at the psychological level and provide corresponding care according to psychiatrists’ assessment of the psycho-psychiatric symptoms of patients. For patients with the psychological distress of clinical diagnosis, treatment by psychiatrists is needed to improve the psycho-spiritual pain; for patients with psychological distress but the distress is of no clinical significance, psychological support and patient education are suggested to reduce the fear and anxiety for disease progression.

## 7 Limitations

This expert consensus has some limitations. Due to insufficient clinical evidence from RCT studies, the recommended clinical application of bone-modifying drugs is based on expert experience, such as the starting time and duration of treatment. There is no high-quality randomized controlled evidence to show whether delayed treatment with bone-modifying drugs has adverse effects on patients. Regarding the duration of therapy, because patients with tumors such as breast cancer have a longer survival time, the recommended treatment duration for breast cancer patients in various guidelines is around 2 years. Considering the shorter survival time of lung cancer and the lack of relevant clinical evidence, the expert group has given a recommended treatment duration of less than 2 years.

In addition, the pivotal studies on bone-modifying drugs cited in this consensus all were conducted ten years ago. After that, although immunotherapy and targeted therapy have greatly improved the prognosis of lung cancer patients, there is no additional high-quality clinical research to prove the combined and synergistic effects of bone-modifying drugs, immunotherapy, and targeted therapy.

## 8 Conclusions

Due to the highest incidence of lung cancer in China, more care should be invested to the early clinical diagnosis and treatment of lung cancer and the accompanying bone metastases. Except for the single disciplinary approach, a comprehensive evaluation based on the MDT pattern is strongly recommended. Because the MDT pattern combines the multi-disciplinary advantages, it could not only treat the primary disease more effectively, prevent or delay the occurrence of SREs, relieve pain, and improve QOL, but also provide psychological support, and thereby comprehensively improve the QOL of this patient population.

## Declaration of competing interest

The authors declare that they have no conflict of interests.
